# Identification of Differentially Expressed Genes and Key Pathways in the Dorsal Root Ganglion After Chronic Compression

**DOI:** 10.3389/fnmol.2020.00071

**Published:** 2020-05-05

**Authors:** Zhanhui Du, Sen Yin, Xiuhui Song, Lechi Zhang, Shouwei Yue, Xiaofeng Jia, Yang Zhang

**Affiliations:** ^1^Department of Physical Medicine & Rehabilitation, Qilu Hospital, Shandong University, Jinan, China; ^2^Heart Center, Qingdao Women and Children’s Hospital, Qingdao, China; ^3^Department of Neurology, Qilu Hospital, Shandong University, Jinan, China; ^4^Department of Neurosurgery, The People’s Hospital of Jimo City, Qingdao, China; ^5^Department of Physical Medicine & Rehabilitation, Suzhou Hospital Affiliated to Nanjing Medical University, Suzhou, China; ^6^Department of Neurosurgery, University of Maryland School of Medicine, Baltimore, MD, United States; ^7^Department of Orthopaedics, Anatomy & Neurobiology, University of Maryland School of Medicine, Baltimore, MD, United States; ^8^Departments of Biomedical Engineering, Anesthesiology and Critical Care Medicine, The Johns Hopkins University School of Medicine, Baltimore, MD, United States

**Keywords:** neuropathic pain, bioinformatic analysis, differentially expressed gene, gene ontology analysis, pathway analysis, KEGG database, CCD

## Abstract

Neuropathic pain (NP) is caused by primary or secondary impairment of the peripheral or central nervous systems. Its etiology is complex and involves abnormal patterns of gene expression and pathway activation. Using bioinformatics analysis, we aimed to identify NP-associated changes in genes and pathways in L4 and L5 dorsal root ganglia (DRG) in a rat model of NP induced by chronic compression of the DRG (CCD). Genome-wide transcriptional analyses were used to elucidate the molecular mechanisms underlying NP. We screened differentially expressed genes (DEGs) 7 days after CCD in comparison with sham-operated controls. Quantitative real-time polymerase chain reaction (RT-qPCR) and western blotting were used to confirm the presence of key DEGs. Kyoto Encyclopedia of Genes and Genomes (KEGG)-pathway analysis of DEGs and global signal transduction network analysis of DEGs were also conducted. The CCD group developed clear mechanical and thermal allodynia in the ipsilateral hind paw compared with the sham group. This comparison identified 1,887 DEGs, with 1156 upregulated and 731 downregulated DEGs, and 123 DEG-enriched pathways. We identified the key candidate genes that might play a role in the development of NP, namely *syndecan 1* (*Sdc1*), *phosphatidylinositol-4,5-bisphosphate 3-kinase*, *catalytic subunit gamma* (*Pi3k*), *Janus kinase 2* (*Jak2*), *jun proto-oncogene, AP-1 transcription factor subunit* (*Jun*), and *interleukin 6* (*IL-6*) by analyzing the global signal transduction network. RT-qPCR and western blot analysis confirmed the microarray results. The DEGs *Sdc1*, *Pi3k*, *Jak2*, *Jun*, and *IL-6*, and the cytokine signaling pathway, the neuroactive ligand-receptor interaction, the toll-like receptor signaling pathway, and the PI3K-Akt signaling pathway may have decisive modulatory roles in both nerve regeneration and NP. These results provide deeper insight into the mechanism underlying NP and promising therapeutic targets for its treatment.

## Introduction

Neuropathic pain (NP; or spontaneous pain) lacks organic causes and usually arises from a primary lesion or dysfunction of the nervous system (Woolf and Mannion, 1999). It has a global incidence of 7–10% (van Hecke et al., 2014). Patients present with varying degrees of mechanical and heat hyperalgesia and sleep and anxiety disorders, which impair their quality of life (Attal et al., 2011; Finnerup et al., 2016). The efficacy of pharmacotherapy for treating NP is limited, owing to undesirable side effects and poor responsiveness (Jacobs, 2017). Although several novel therapies have been developed for NP, the mechanism underlying NP remains unclear. Clarifying the molecular mechanism underlying NP remains an unresolved issue in the field of medical research, and a solution could lead to better clinical management of NP.

Neuronal sensitization is thought to play a key role in the development and maintenance of NP (Teixeira et al., 2015). Inflammation is inevitable during this process, and allodynia and hyperalgesia are common features. Gene expression is altered in the spinal cord and dorsal root ganglion (DRG), as evidenced by inconsistent results in different animal models of peripheral nerve injury (Wang et al., 2017; Liu et al., 2018; Zhao et al., 2020). The chronic compression of the DRG (CCD) model mimics clinical radicular pain, such as spontaneous pain, hyperalgesia, and allodynia. In comparison with other models, there are distinct properties of the CCD model, such as maintenance of peripheral input, absence of motor disorders, and ectopic discharge of neurons. Studies on CCD are essential to improve understanding of comprehensive neuropathic pain; they will also provide an opportunity to compare the results with other models.

The DRG has been proven to be involved in NP and nerve regeneration as a modulatory locus, and it may be the most important therapeutic target. Several studies have reported that the expression of ion channels, chemokines, and receptors changed after CCD (Oh et al., 2001). However, these studies focused on individual genes and failed to provide insight into the overall changes and the networks between the target genes. New insights have been achieved into the diagnosis and treatment of several diseases, with the development of high-throughput gene screening technology such as microarrays. The use of microarrays has rendered gene expression profiling a robust and straightforward process for studying the molecular features of diseases at the systemic level. Therefore, research on the molecular mechanism of NP based on the CCD model using microarrays may be useful for the development of appropriate clinical therapies for NP. This study aimed to explore the central genes that are dysregulated and the signaling pathways that are activated in a rat CCD model of NP using bioinformatics analysis.

## Materials and Methods

### Experimental Animals

A total of 34 Wistar rats (adult male, weighing 180–200 g) were purchased from the Experimental Animal Center of Shandong University and housed in a room with pathogen-free air at 20 ± 2°C, with two rats in a cage, at a 12-h light/dark cycle. Water and food were available *ad libitum*. The experimental operation began on the 8th day so that the animals were habituated to their environment. The rats were divided into the sham and CCD groups randomly, in which 18 rats were used for microarray analysis (eight in the CCD group; 10 in the sham group) and 16 rats were used for behavioral studies and molecular experiments (eight in each group). Each behavioral study began 10 min after the animal entered the experimental environment. All experimental procedures were approved by the Animal Care and Use Committee of Shandong University.

### CCD Model

CCD was induced as described earlier (Jia et al., 2018). In summary, after the rats were anesthetized, the paraspinal muscles were separated to unilaterally expose the transverse processes and intervertebral foramina of L4 and L5. Two L-shaped stainless steel rods (diameter = 0.63 mm and length = 4 mm) were implanted into the intervertebral foramina of L4 and L5 at an oblique angle of 30° with the spinal column to compress the DRGs. The other end of the bar was maintained out of the intervertebral foramen. Rats in the sham-operated group underwent the same procedure without steel rod insertion. Those exhibiting autophagy, tactile sensory deficiencies, or disabilities were excluded. The rats were administered deep anesthesia with isoflurane and were sacrificed after behavioral testing on the 7th-day post-surgery. The L4 and L5 DRG were then harvested for microarray analysis and validation tests.

### Nociceptive Behavioral Testing

Mechanical hypersensitivity was determined by the same experimenter before the procedure and on the 1st, 3rd, 5th, and 7th days after CCD induction, respectively (Qu et al., 2016). The paw withdrawal threshold was automatically recorded by the BEM-404 mechanical analgesia tester [Chinese Academy of Medical Sciences (CAMS), Beijing, China] and the cutoff force was set at 50 g. Tests were started 10 min after the rats entered the test environment. A rigid tip was applied perpendicularly to the mid-plantar surface of the hind paw. Brisk withdrawal or paw flinching was considered a positive response. The number displayed on the digital monitor was recorded as the paw withdrawal mechanical threshold (PWMT). Three successive stimuli were applied. The mean of three successive measurements was recorded as the PWMT of each animal.

Thermal paw withdrawal latency (TPWL) was measured using the BME-410C thermal analgesia tester (CAMS) as described previously (Jia et al., 2018). The light source was a 12 V/10 W halogen lamp and the thermal intensity was set at 60°C. Radiant light was focused on the center of the hind paw at about 1 cm, and TPWL was recorded when the rat lifted or licked the hind paw. A 25-s cutoff period was used to avoid tissue damage. The test was repeated three times for each rat with a 5-min interval between successive measurements; the mean of three successive measurements was recorded as the TPWL of each animal.

### Total RNA Extraction and Microarray Assay

One sample for each group (sham and CCD) was collected by pooling the L4 and L5 DRG of two rats. Total RNA was extracted and purified with an RNeasy Mini Kit (Qiagen, Hilden, Germany) according to the manufacturer’s instructions. The quality and amount of RNA were measured using NanoDrop 2000 (Thermo, USA). mRNA expression profiling was performed using Affymetrix Clariom D Rat array (Affymetrix; Thermo Fisher Scientific Inc., Waltham, MA, USA), which contains 46,800 gene-level probe sets. GeneChips were washed, stained, and subsequently scanned using the 3000 7G GeneChip Scanner. Raw data were analyzed using the Robust Multichip Analysis (RMA) algorithm with the Affymetrix Expression Console Software (version 1.2.1). The values were presented as log_2_ RMA signal intensities. The microarray data discussed in this article have been submitted to the NCBI Gene Expression Omnibus and can be accessed through the GSE accession number (GSE145222).

### Data Analysis

Differentially expressed genes (DEGs) were identified using the random variance model *t*-test and false discovery rate (FDR) analysis for comparisons between two groups. DEGs were considered to be present if *p* < 0.05 and the absolute value of fold change (FC) >2. DEGs were considered up or down-regulated if there was at least a 2-fold change in the positive or negative direction, respectively. Hierarchical clustering was performed using Cluster Treeview software (Palo Alto, CA, USA) to observe the DEG-expression pattern.

Gene ontology (GO) analysis was used to classify DEGs into different hierarchical categories based on the biological process and molecular function and reveal the gene regulatory network. Pathway analyses of DEGs were determined according to the Kyoto Encyclopedia of Genes and Genomes (KEGG) database. The significance of the pathway was determined using Fisher’s exact test. The *P*-value was corrected by FDR analysis and a *p*-value < 0.05 was considered statistically significant.

A global signal transduction network was constructed using Cytoscape V3.6.0 based on the KEGG database to illustrate the inter-gene signaling of DEGs and discover the core genes playing an important role in this network. The nodes and lines in the network graphs represented the genes and the interactions between the nodes, respectively, such as inhibition of phosphorylation. “In-degree,” “out-degree,” or “degree” represented the number of links of a single node with its upstream genes, downstream genes, or all binding genes, respectively. The hub gene was selected based on the degrees. Genes with higher degrees had a more crucial position in the network.

### Real-Time Polymerase Chain Reaction Analysis

Quantitative real-time polymerase chain reaction (RT-qPCR) was performed as described previously (Du et al., 2018). The total RNA was extracted and reverse-transcribed into cDNA. qPCR was performed on an iQ5 Real-Time PCR cycler (Bio-Rad, Hercules, CA, USA). All samples were normalized to Gapdh values. Data are shown as fold changes (2^−ΔΔCT^). The primer sequence used is shown in Table 1.

**Table 1:** Primers used in Quantitative real-time polymerase chain reaction (RT-PCR) analysis.

Gene	Sequence 5′-3′
Pi3k	F: TCTCTGGACCTGTGCCTTCT
	R: GCCTGTCACCTATCCCAAGA
Jak2	F: AGATATGCAAGGGCATGGAG
	R: GTCAAGGATTCGGGAGCATA
Gng7	F: ACTTTGTCTCCGAAGCCTGA
	R: AGAAAATGGCCACAGTCCAC
Jun	F: CAGGTGGCACAGCTTAAACA
	R: CGCAACCAGTCAAGTTCTCA
IL6	F: CCGGAGAGGAGACTTCACAG
	R: ACAGTGCATCATCGCTGTTC
Met	F: CAGCGGCAATTCTAGACACA
	R:CTGAAGCTGCTTGTCACTCG
Pdgfra	F: CACAATAACGGGAGGCTGGT
	R: GTTCTGACGTGGCTTTCAAGG
Camk2b	F: GGAGTCAAGCCCCAGACAAAC
	R: GTGTTGGTGCTGTCGGAAGATT
Ptgs2	F: GTGGGATGACGAGCGACTG
	R: CCGTGTTCAAGGAGGATGG
Sdc1	F: ACCATCAGCCTCCAAGTGTG
	R: TGAAGTCTTGTTCTCCAGAGCC
Gapdh	F: AGGTCGGAGTCA ACGGATTTGGT
	R: CATGTGGGCCATGAGGTCCACCAC

*Pi3k, phosphatidylinositol-4,5-bisphosphate 3-kinase catalytic subunit gamma; Jak2, Janus kinase 2; Gng7, G protein subunit gamma 7; Jun, Jun proto-oncogene, AP-1 transcription factor subunit; IL6, interleukin 6; Met, MET proto-oncogene, receptor tyrosine kinase; Pdgfra, platelet-derived growth factor receptor alpha; Camk2b, calcium/calmodulin-dependent protein kinase II beta; Ptgs2, prostaglandin-endoperoxide synthase 2; Sdc1, syndecan 1; F, forward; R, reverse*.

### Western Blotting

Total proteins were extracted from tissues using RIPA Lysis Buffer (Beyotime, Haimen, Jiangsu, China). Equal amounts of the protein were separated using sodium dodecyl sulfate-polyacrylamide gel electrophoresis and transferred to a polyvinylidene fluoride membrane, followed by overnight incubation with primary antibodies at 4°C. Primary antibodies against the following proteins were used: Pi3k (1:300, 20584-1-AP, Proteintech), Jak2 (1:4,000, ab108596, Abcam), Gng7 (1:800, GTX65584, GeneTex), Jun (1:1,500, 66313-1-Ig, Proteintech), IL-6 (1:1,000, ab9324, Abcam), Met (1:1,500, ab51067, Abcam), Pdgfra (1:1,000, ab203491, Abcam), Camk2b (1:1,000, ab34703, Abcam), Ptgs2 (1:1,000, ab15191, Abcam), Sdc1 (1:1,000, ab128936, Abcam), and Gapdh (1:10,000; SAB2100894; Sigma–Aldrich; Merck KGaA). Target protein expression was normalized to Gapdh expression.

### Statistical Analysis

All data are presented as mean ± SEM. The repeated measures two-way ANOVA test was used for the statistical analysis of the sham and CCD groups using SPSS 20.0 software (IBM Company, Armonk, NY, USA). Significant GO categories and pathways were selected based on Fisher’s exact test, and all *p*-values were FDR adjusted *p*-values. A *p*-value < 0.05 was considered statistically significant.

## Results

### PWMT and TPWL Changes After CCD Operation

**Figure 1:**
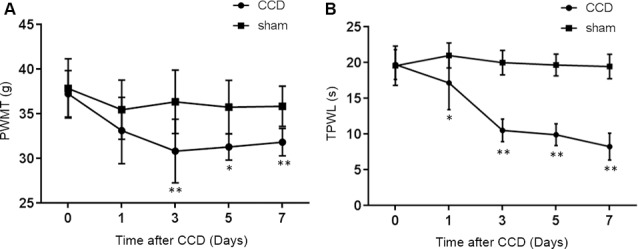
PWMT and TPWL changes after chronic compression of the DRG (CCD) operation. **(A)** The PWMT decreased significantly 3rd, 5th, and 7th days after CCD surgery when compared with the sham group. **(B)** Compared to the sham group, TPWL decreased from the 1st to 7th-day post-surgery; *n* = 8 in both groups. PWMT, the paw withdrawal mechanical threshold; TPWL, thermal paw withdrawal latency. *P < 0.05 and **P < 0.01 compared with the sham group.

The CCD group developed clear mechanical allodynia in the ipsilateral hind paw, compared with the sham group (Figure 1A). The PWMT decreased significantly 3rd (*p* = 0.008), 5th (*p* = 0.002), and 7th (*p* = 0.001) days after CCD operation (*n* = 8 in each group).

Thermal hyperalgesia was determined using the TPWL test. As indicated in Figure 1B, the TPWL decreased significantly (similar to the PWMT) from the 1st to 7th-day post-surgery (all *p* ≤0.001), when compared to the sham-operated rats (*n* = 8 in each group).

### Analysis of mRNA With Differential Expression

**Figure 2:**
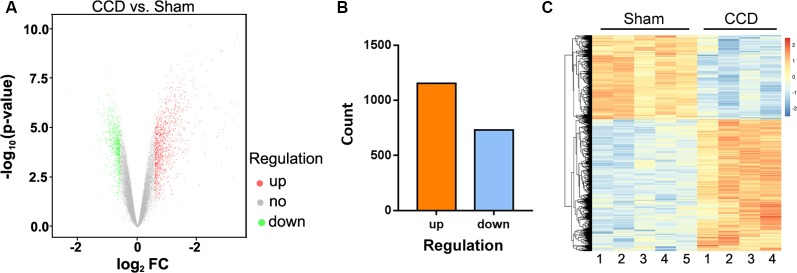
DEGs between the CCD group and the sham group. **(A)** Volcano plot of the −log_10_(*p*-value) against the log_2_FC of each gene. Red dot indicates an upregulated DEG, whereas green dot indicates a downregulated gene. Gray indicates that there is no statistically significant difference in gene expression. **(B)** There were 1,887 DEGs identified between the CCD group and the sham group. Of these genes, 1,156 were upregulated and 731 were downregulated. **(C)** Hierarchical clustering shows the DEGs between the two groups. The number at the bottom represents the name of the samples. The color scale at the top right represents the normalized expression data, which represents the degree of gene clustering. The upregulated genes are indicated in red, and the downregulated genes are indicated in blue. DEGs, differentially expressed genes; CCD, chronic compression of the dorsal root ganglion surgery group; FC, fold change; sham, compression of the dorsal root ganglion sham group. The *p*-value was corrected by false discovery rate (FDR).

Microarray analysis identified 1,887 differentially expressed genes (DEGs) in comparing the CCD and sham groups. A volcano plot was applied to visualize the genes identified (Figure 2A). Among all these genes, 1,156 were upregulated and 731 were downregulated (Figure 2B). Figure 2C shows a heatmap of DEG expression; it demonstrates that the mRNA expression profiles of the two groups were distinct (*n* = 4 in the CCD group, *n* = 5 in the sham group).

### GO-Term Enrichment Analysis of DEGs

**Figure 3:**
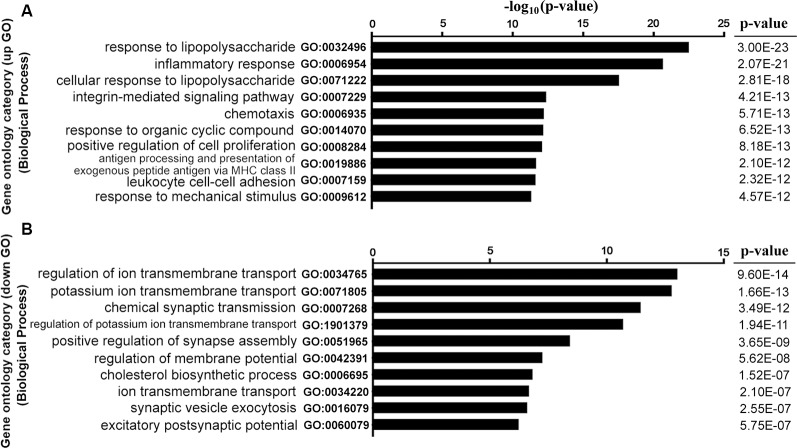
Functional enrichment analysis for DEGs. GO terms enriched by upregulated (upGOs) and downregulated genes (downGOs) are shown as **(A)** and **(B)**, respectively. The horizontal axis represents the value of −log_10_ (*p*-value), and the vertical axis represents the category of GO biological processes. *P*-value was adjusted by FDR. GO, gene ontology. BP, biological process.

GO analysis was performed to explore the main cell functions of DEGs. The top five GO terms among the CCD-upregulated genes (upGOs) were as follows: response to lipopolysaccharide, inflammatory response, cellular response to lipopolysaccharide, integrin-mediated signaling pathway, and chemotaxis (Figure 3A). The GO terms for CCD-downregulated genes (downGOs) included regulation of ionic transmembrane transport, potassium ion transmembrane transport, regulation of potassium transmembrane transport, chemical synaptic transmission, and positive regulation of synapse assembly (Figure 3B).

### KEGG-Pathway Analysis of DEGs

**Figure 4:**
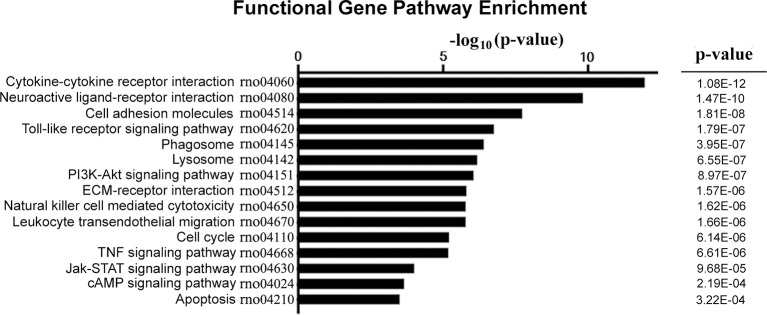
KEGG pathway enrichment analysis for DEGs. The vertical axis on the left side represents the name of the pathway, and the horizontal axis at the top represents the value of −log_10_ (*p*-value). *P*-value was adjusted by FDR. KEGG, Kyoto Encyclopedia of Genes and Genomes.

The DEGs were subjected to pathway analysis based on the KEGG database. The analysis revealed 123 enriched pathways for both upregulated and downregulated DEGs. As shown in Figure 4, several important pathways were altered in our NP model, including cytokine-cytokine receptor interaction, neuroactive ligand-receptor interaction, cell adhesion molecules, toll-like receptor signaling pathway, phagosome, lysosome, PI3K-Akt signaling pathway, cell cycle, TNF signaling pathway, Jak-STAT signaling pathway, and apoptosis.

### Global Signal Transduction Network Analysis of DEGs

**Figure 5:**
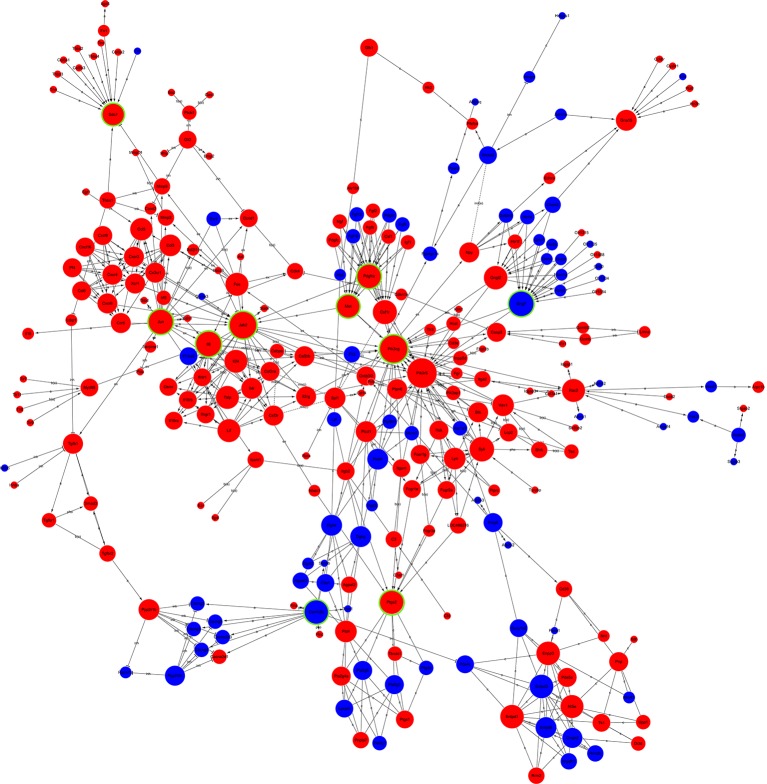
Global signal transduction network of DEGs. The red node represents an upregulated gene, and the blue node represents the downregulated gene. The lines exhibit the interaction between the genes. The size of the node indicates the degree of interacting with other genes. The more important the genes are, the larger the node is. Nodes with green rings were the genes that were selected to be experimentally validated.

The candidate genes that could play a key role in the development of NP were screened using global signal transduction network analysis. As illustrated in Figure 5, the high-degree genes were: *phosphatidylinositol-4,5-bisphosphate 3-kinase, catalytic subunit gamma* (*Pi3k*), *Janus kinase 2* (*Jak2*), *G protein subunit gamma 7* (*Gng7*), *Jun proto-oncogene* aka *AP-1 transcription factor subunit* (*Jun*), *interleukin 6* (*IL-6*), *MET proto-oncogene* aka *receptor tyrosine kinase* (*Met*), *platelet-derived growth factor receptor alpha* (*Pdgfa*), *calcium/calmodulin-dependent protein kinase II beta* (*Camk2b*), *prostaglandin-endoperoxide synthase 2* (*Ptgs2*), and *syndecan 1* (*Sdc1*). *Gng7* and *Camk2b* were downregulated while the others were upregulated (Table 2).

**Table 2:** Ten key genes identified by global signal transduction network analysis.

Gene symbol	Offical full name	Style	Degree	Indegree	Outdegree	*p*-value	Fold change
Pi3k	phosphatidylinositol-4,5-bisphosphate 3-kinase catalytic subunit gamma	up	30	24	6	0.003	1.79
Jak2	Janus kinase 2	up	26	23	3	0.0008	1.75
Gng7	G protein subunit gamma 7	down	18	14	4	0.002	−1.65
Jun	Jun proto-oncogene, AP-1 transcription factor subunit	up	18	2	16	0.0005	1.89
IL6	interleukin 6	up	16	5	11	0.001	2.24
Met	MET proto-oncogene, receptor tyrosine kinase	up	13	10	3	7.321E-0.5	2.34
Pdgfra	platelet derived growth factor receptor alpha	up	13	10	3	0.0006	1.65
Camk2b	calcium/calmodulin-dependent protein kinase II beta	down	12	0	12	0.0006	−1.51
Ptgs2	prostaglandin-endoperoxide synthase 2	up	12	10	2	0.014	2.51
Sdc1	syndecan 1	up	12	12	0	8.327E-0.5	1.96

*P-value was corrected by FDR*.

**Figure 6:**
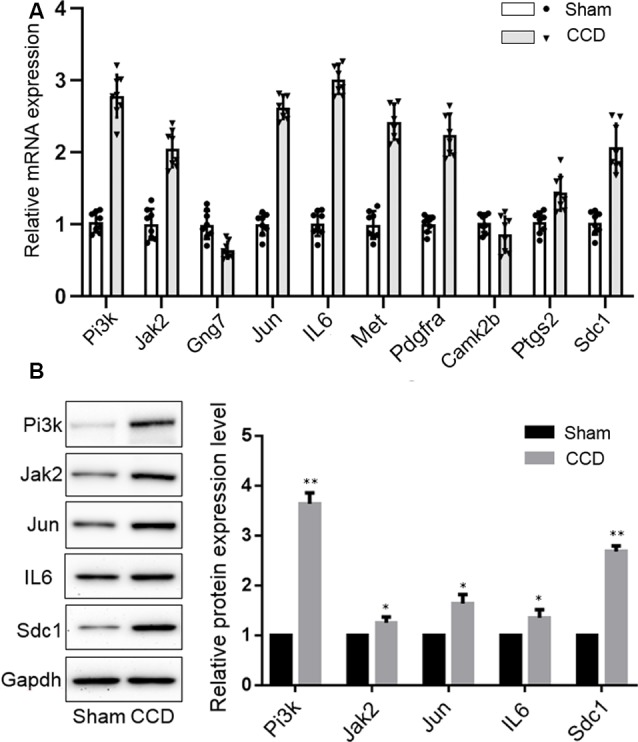
Experimental validation of key genes. **(A)** Reverse-transcription quantitative chain reaction was performed to detect the mRNA expression of 10 key genes. **(B)** Western blotting validation of the protein expression changes of key genes in the CCD and sham group. Gapdh was used as a loading control. *p < 0.05 and **p < 0.01 compared with the sham group.

qPCR was performed to validate the mRNA levels of the ten DEGs. *Gng7* and *Camk2b* were found to be downregulated and the remaining genes were upregulated (Figure 6A), supporting the microarray results. Moreover, western blotting confirmed that the protein levels of *Pi3k*, *Jak2*, *Jun*, *IL-6*, and *Sdc1* were markedly higher in the CCD group than in the sham group (Figure 6B). However, there was no difference in protein expression of *Gng7*, *Met*, *Pdgfra*, *Camk2b*, and *Ptgs2* (data are not shown in the figure).

## Discussion

NP is a commonly encountered neurological condition wherein patients feel pain in the absence of external stimuli; this is termed “spontaneous pain.” Alterations in gene and protein expression characterize the development and maintenance of NP (Gold and Gebhart, 2010; Wang et al., 2017). In the present study, we analyzed the mRNA expression patterns after CCD in the L4 and L5 DRG, which are important sites of pain regulation, synaptic plasticity, and NP treatment. Comparisons of sham-operated and CCD rats revealed expression changes in 1,887 genes. GO term enrichment analysis-term enrichment analysis of DEGs showed that the top GO terms among upGOs included a response to inflammation, integrin-mediated signaling pathway, and chemotaxis; those among downGOs included regulation of ionic transmembrane transport.

Recent bioinformatic analysis has explored the genetic processes and molecular mechanisms underlying NP, and several DEGs have been identified (Wang et al., 2017; Liu et al., 2018; Yang et al., 2018; Tang et al., 2020). Cobos et al. (2018) obtained a PPI network consisting of 31 nodes and 442 edges, which revealed certain key signaling molecules such as ATF3, JUN, BDNF, and MAPK1/3 in the sciatic nerve injury (SNI) model. Using the same dataset, Tang et al. (2020) identified additional 10 major DEGs, such as *Npy, Atf3*, and *Gpr151*, that were predominantly associated with cytokine-cytokine receptor interactions and the p53 signaling pathway. CCL2, NF-κB1, and C1Q in the dorsal horns (Wang et al., 2017), p53 and active caspase-3 in the DRG (Gao et al., 2018), Ccl3, Atf3, and Tgif1 in the spinal cord (Zhang and Yang, 2017) were separately considered to contribute to the generation of NP after SNI, chronic constriction injury (CCI), and spinal cord injury (SCI). Our study showed that *Jun* and *IL-6* may evoke the progression of NP after CCD; this was consistent with other bioinformatics analysis (Liu et al., 2018; Yang et al., 2018; Tang et al., 2020). Therefore, the genetic targets differed with the pathological conditions and analytical methods. The CCD model mimics the effects of protrusion of an intervertebral disc through the injuring of both sensory neurons and spinal nerves; this distinguishes it different from other animal models. Moreover, the steel rods used in the protocol could potentially induce sterile inflammation and damage spinal segmental vessels passing through the intervertebral foramina. Hence, spinal compression and the accompanying inflammation may contribute to the behavioral and physiological consequences of CCD and greater complexity in the local microenvironments (Yang et al., 2018). This is consistent with our observation that the upGO terms enriched by DEGs after CCD were the inflammatory response and integrin-mediated signaling pathway, that were associated with mechanotransduction.

Meanwhile, the main downGOs categories of DEGs were the regulation of ion transmembrane transport, potassium ion transmembrane transport, and regulation of membrane potential. It is well known that potassium ion channels, inward rectifying potassium channels (Kir) in particular, maintain strongly negative resting membrane potential (Rosenhouse-Dantsker, 2019). Thus, downGOs of potassium ion transmembrane transport and regulation of membrane potential may be associated with neuronal somal hypeand peripheral axotomyrexcitability and spontaneous action potentials after CCD. However, Gao et al. (2018) reported that the DEGs associated with the regulation of transmembrane transport and regulation of membrane potential were upregulated after CCI. This further demonstrates the importance of research in different pathological conditions. Interestingly, the downGOs included the cholesterol biosynthetic process. It has been demonstrated that many ion channels including Kir channels, in particular, are modulated by cholesterol (Rosenhouse-Dantsker, 2019). Therefore, our findings provide a new insight into the understanding of the mechanism of cellular excitability and ion channel rearrangements after CCD.

We found that the expression of *Sdc1* was significantly increased 7 days after CCD. Sdc1 is expressed in C-fiber sensory neurons distributed in peripheral and central axons. It was demonstrated that *Sdc1* could reduce neurite extension in primary cultured DRG neurons in both, the proximal region and distal regions of the injured sciatic nerve (Murakami et al., 2015). Sciatic nerve axotomy and infraorbital nerve axotomy induced *Sdc1* in primary sensory neurons, which may contribute to the development of abnormal nociception (Couchman, 2003). Moreover, *Sdc1* expression plays a functional role in nerve regeneration and synaptic plasticity in injured primary sensory neurons (Edwards and Hammarlund, 2014; Bray et al., 2019). The increased expression of *Sdc1* may have multiple functions in nerve regeneration and the development of abnormal nociception after CCD; this needs further research.

As shown in Figure 5, the expression of *Jun* and *IL-6* increased significantly 7 days after CCD; this was consistent with findings from previous studies (Ding et al., 2016; Zhao et al., 2020). *Jun* is expressed at lower levels in normal nervous systems; however, it is upregulated in response to harmful stimuli such as dorsal column transection, root rhizotomy, and peripheral axotomy, et cetera (Kenney and Kocsis, 1998; Li et al., 2013). Notably, the c-Jun N-terminal kinase (JNK) signaling pathway is essential for chronic inflammatory pain (Sanna and Galeotti, 2018). Inhibition of JNK signaling was effective in relieving inflammatory pain and NP in several animal models (Li et al., 2017). The upregulation of *Jun* also showed protective and nerve-regenerative roles in DRG and Schwann cells in a nerve injury model (Yang et al., 2012; Zhao et al., 2014). JNK inhibitors may enhance the expression of activated transcription factor 3 and decrease the expression of multiple neurotrophic factors secreted by the DRG (Tu et al., 2016). Thus, an increase in the expression of *Jun* may be correlated with post-CCD pain and nerve regeneration.

The role of *IL-6* in nociceptive processing and regulation of pathological pain is well established (Schaper and Rose-John, 2015). *IL-6* was shown to induce the JAK-mediated phosphorylation of STAT proteins (Schaper and Rose-John, 2015) and participate in the later maintenance of NP by activating the JAK/STAT3 signaling pathway (Ding et al., 2016). Although the pain caused by *IL-6* is subjectively unpleasant, several studies suggest that IL-6 is beneficial to the nervous system. *IL-6* is rapidly expressed in the retina and promotes the regeneration of the optic nerve by activating the JAK/STAT3 and PI3K/AKT signaling pathways following optic nerve injury (Leibinger et al., 2013). In general, *IL-6* has detrimental and beneficial effects on the nervous system.

The upregulation of JAK/STAT3 signaling has been observed after nerve injury in animal models of neuropathic pain (Dominguez et al., 2008, 2010). Activation of the JAK/STAT3 cascade is induced by both pro-nociceptive (IL-6) and anti-nociceptive (IL-10) factors. These two forms of activation lead to the transcription of different pools of genes affecting the state of polarization of the microglia. The PI3K/AKT signaling pathway is also involved in the IL-6–mediated regulation of pathological pain (Ding et al., 2018). Plantar incisions have been found to induce time-dependent activation of the PI3K/Akt pathway in the spinal cord and DRG (Xu et al., 2014). As shown in Figure 4 and Table 2, the key candidate genes *JAK*, *IL-6*, *Jun*, and *PI3K* are vertices of a global signal transduction network and act synergistically; this is consistent with the findings of other studies. They are also involved in a complex physiological response that involves the termination of inflammation, promotion of the M2 state of microglial polarization, and synaptic plasticity (Nicolas et al., 2013; Xue et al., 2014).

Our study has several limitations. First, tissue samples obtained from rats differ from those of humans and may not show the same target genes after nerve injury. Second, we detected the DEGs 7 days after CCD, which may reveal genes involved in the development, but not in the maintenance of NP. Third, the analysis of a larger number of tissue samples is required to improve the reliability of these results.

In conclusion, we found that *Sdc1*, *Pi3k*, *Jak2*, *Jun*, *IL-6*, and the signaling pathways cytokine-cytokine receptor interaction, neuroactive ligand-receptor interaction, toll-like receptor signaling pathway, and PI3K-Akt signaling pathway have key roles in both nerve regeneration and NP. These results may provide deeper insight into the understanding of the mechanism of NP after CCD, and help to identify promising therapeutic targets. Further studies are needed to confirm the role of cytokines and signaling pathways in the development of neuropathic pain, both individually and in cooperation.

## Data Availability Statement

The microarray data discussed in this article have been submitted to the NCBI Gene Expression Omnibus and can be accessed through the GSE accession number (GSE145222).

## Ethics Statement

Procedures were approved by the Chinese Institutional Animal Care Committee of Shandong University.

## Author Contributions

ZD, SYi, and XS performed the experiments and analyzed the data. YZ and LZ wrote the manuscript. XJ revised the manuscript and provided critical appraisal. SYu and YZ conceived the original idea and designed the experiments for this study. All authors read and approved the final manuscript.

## Conflict of Interest

The authors declare that the research was conducted in the absence of any commercial or financial relationships that could be construed as a potential conflict of interest.
